# Amantadine ER (Gocovri^®^) Significantly Increases ON Time Without Any Dyskinesia: Pooled Analyses From Pivotal Trials in Parkinson's Disease

**DOI:** 10.3389/fneur.2021.645706

**Published:** 2021-03-26

**Authors:** Robert A. Hauser, Ryan R. Walsh, Rajesh Pahwa, Dustin Chernick, Andrea E. Formella

**Affiliations:** ^1^Department of Neurology, University of South Florida, Tampa, FL, United States; ^2^Muhammad Ali Parkinson Center at Barrow Neurological Institute, Phoenix, AZ, United States; ^3^Department of Neurology, University of Kansas Medical Center, Kansas City, KS, United States; ^4^Adamas Pharmaceuticals, Inc., Emeryville, CA, United States

**Keywords:** amantadine, dyskinesia, Parkinson's disease, treatment, Gocovri, motor complications, home diary, OFF

## Abstract

**Background:** Clinical trials for antiparkinsonian drugs aimed at managing motor complications typically use patient diaries to divide levodopa-induced dyskinesias (LID) into “troublesome” and “non-troublesome” categories. Yet, given the choice, most patients would prefer to live without experiencing any dyskinesia. However, the concept of evaluating time spent ON *without* any dyskinesia as an outcome has never been tested. We conducted analyses of pooled Gocovri pivotal trial data in order to evaluate the extent to which Gocovri increased the time PD patients spent ON *without* dyskinesia (troublesome or non-troublesome), beyond its already identified improvement in reducing troublesome dyskinesia.

**Methods:** Patients enrolled in phase 3 trials (EASE LID [NCT02136914] or EASE LID 3 [NCT02274766]) recorded time spent in the following PD diary states at baseline and Week 12 (endpoint): asleep, OFF, ON with troublesome dyskinesia, ON with non-troublesome dyskinesia, and ON *without* dyskinesia. Mixed model repeated measures analyses with estimated Cohen D effect sizes were performed on the modified intent to treat population to evaluate changes in time spent in these states.

**Results:** Patients randomized to receive Gocovri showed an increase in ON time *without* dyskinesia and corresponding decreases in ON time with dyskinesia and OFF time vs. placebo. Treatment effects were statistically significant for Gocovri vs. placebo starting at Week 2 and were sustained until Week 12. On MMRM analysis at Week 12, patients in the Gocovri group showed an adjusted mean ± SE increase over placebo of 2.9 ± 0.6 h in ON time *without* dyskinesia (Cohen D effect size 0.79) and an adjusted mean ± SE decrease of −1.9 ± 0.6 h in ON time with dyskinesia (troublesome + non-troublesome) (Cohen D effect size 0.49), that included a −1.5 ± 0.4 h placebo-adjusted reduction in ON time with troublesome dyskinesia and a −0.6 ± 0.4 h reduction in ON time with non-troublesome dyskinesia. OFF time was reduced by −1.0 ± 0.3 h compared to placebo.

**Conclusions:** Gocovri treatment more than doubled the daily time patients spent ON *without* dyskinesia. These results suggest that the Gocovri treatment effect was driven by a reduction in overall motor complications including ON time with both troublesome and non-troublesome dyskinesia as well as time spent OFF.

## Introduction

Levodopa-induced dyskinesias (LID) are a source of disability, discomfort, and stigma for many people with Parkinson's disease (PD) (1–6). The incidence of LID increases by about 10% per year of disease (7, 8), and can affect daily activities such as walking, dressing, eating and leisure activities. (9) At present, most adjunctive PD treatments are dopaminergic (dopamine agonists, MAO-B and COMT inhibitors) and are initiated to manage motor fluctuations. However, since 40–60% of patients with motor fluctuations also experience LID (10, 11), there is limited room in such patients to increase dopaminergic-based therapies without exacerbating LID, and alternative approaches are necessary.

Amantadine, which has activity at NMDA receptors, is currently the only antiparkinsonian drug with established efficacy against LID (12, 13). Available since the 1960's, immediate-release amantadine preparations have been associated with inconsistent antidyskinetic efficacy at lower doses and tolerability concerns at higher doses. An extended-release formulation, Gocovri (Gocovri^®^, Adamas Pharmaceuticals, Emeryville, CA), was developed to improve amantadine's pharmacokinetic profile, with the aim of enhancing the antidyskinetic efficacy without compromising tolerability or needing to adjust the dopaminergic pharmacologic regimen. Recent studies with Gocovri show that treatment not only reduces LID, but also has a significant effect in reducing OFF time and increasing ON time without troublesome dyskinesia (14, 15).

While clinical trials based on patient reported diaries, typically divide dyskinesia into “troublesome” and “non-troublesome” categories, given the choice, most patients would prefer to live without experiencing any dyskinesia (1, 16–18). Thus, we conducted analyses of pooled Gocovri pivotal trial data in order to evaluate the extent to which Gocovri decreased ON time with dyskinesia (troublesome + non-troublesome) and increased the time PD patients spent ON *without* any dyskinesia, beyond its already identified improvement in reducing ON time with troublesome dyskinesia.

## Methods

### Study Designs and Participants

The efficacy and safety of Gocovri as an antidyskinetic agent in PD was established in two, similarly designed phase 3 pivotal trials. The full methodologic details of these trials have been previously published:

1. A randomized, double-blind, placebo-controlled, 25-week clinical trial (NCT02136914) conducted in 44 sites in the United States and Canada (14).
2. A randomized, double-blind, placebo-controlled, 12-week trial (NCT02274766) conducted at 39 sites in the United States and Western Europe (Germany, France, Spain, and Austria) (15).

Briefly, patients (aged 30–85 years old) in both trials were required to be experiencing ≥1 h/day (2 half-hour intervals) of ON time with troublesome dyskinesia between 9 am and 4 pm, on the 2 days preceding treatment initiation, as documented by entries in PD home diaries. Patients' dyskinesia was required to be causing at least mild functional impairment, as documented at screening and baseline by a score ≥2 on item 4.2 of the Movement Disorder Society–Unified Parkinson's Disease Rating Scale (MDS-UPDRS) (19). Enrolled patients were randomized in a 1 : 1 ratio to double-blind Gocovri or placebo once daily at bedtime. Gocovri was initiated at 137 mg/day (corresponding to 170 mg of amantadine HCl) for the 1st week and titrated to 274 mg/day (corresponding to 340 mg of amantadine HCl) thereafter. Levodopa preparations, which had to be administered ≥3 times daily for eligibility, and all other antiparkinsonian medications were to be unchanged for ≥30 days prior to screening and during study participation.

### Efficacy Outcomes and Analyses

The primary efficacy outcome measure in both studies was change from baseline in the Unified Dyskinesia Rating Scale (UDysRS) total score, as assessed at week 12 (14, 15). Relevant to the present analyses, as a key secondary outcome, all patients completed home diaries for the two consecutive days prior to each scheduled visit, in which they categorized their predominant motor state during each half-hour interval of the 24-h day as: ON without dyskinesia, ON with non-troublesome dyskinesia, ON with troublesome dyskinesia, OFF, or asleep (20). Patients and caregivers received training on how to use the diaries during the screening period, and concordance with the diary was confirmed during the baseline period. Diaries with ≥4 missing entries (i.e., missing 2 h) per day were considered unevaluable for analysis. Otherwise, missing data were imputed by assigning the 30 min of each missing interval, in equal portions of 15 min each, to the responses of the immediately preceding and subsequent completed (non-missing) intervals.

Pooled analysis of the two trials was planned prior to conducting the studies contingent on positive results for the primary and key secondary endpoints in each individual trial. Analyses were performed for the modified intent-to-treat (mITT) population, comprising all randomized patients who were exposed to the study drug and provided ≥1 postbaseline assessment. However, as there was no *a-priori* plan for overall control of the Type I error in the pooled analyses for these endpoints, results are considered exploratory. Data integrated from the individual studies were not modified for the integrated analyses in the pooled trials. Reduction in ON time with dyskinesia (troublesome + non-troublesome) with Gocovri (274 mg) vs. placebo was a specified analysis in the statistical plan for data pooling, and was evaluated at Weeks 2, 8, and 12, using mixed effect model repeat measurement (MMRM) analyses.

Additionally, we performed exploratory analyses to determine the increase in ON time *without* dyskinesia using the same MMRM model for other diary states (21) including change from baseline as the dependent variable, and baseline value as a covariate with categorical effects for treatment group, study, and visit (Weeks 2, 8, and 12), and the interaction between treatment group and visit. We also estimated effect sizes [Cohen D] for the Gocovri-placebo treatment difference for both decrease in total dyskinesia (troublesome + non-troublesome) and increase in ON time *without* dyskinesia, using the ratio of the least square (LS) mean treatment difference and the square root of the variance parameter obtained from the MMRM model at the given study week.

To better understand magnitude of patient response, we analyzed the proportion of patients who reduced their ON time with dyskinesia (troublesome + non-troublesome) by 25, 50, 75, and 100%. Treatment differences for these responder rates were analyzed using the Farrington-Manning (FM) score test. To provide a corresponding threshold-type analysis for ON time, we calculated the proportion of patients, at baseline and Week 12, spending at least 25, 50, 75, and 100% of their waking day ON without troublesome dyskinesia and also *without* any dyskinesia (troublesome or non-troublesome). Statistical significance vs. placebo was tested using the Cochran–Mantel–Haenszel test based on movement of patients across these percentage thresholds (based on degree of worsening or improvement).

All analyses were set at a two-sided, 5% significance level and were performed using SAS version 9.4 (SAS Institute Inc., Cary, North Carolina).

### Standard Protocol Approvals, Registrations, and Patient Consents

Both studies (14, 15) were conducted in compliance with the Declaration of Helsinki and International Conference on Harmonization (ICH) Good Clinical Practice (GCP) guidelines for conducting, recording, and reporting trials, as well as for archiving essential documents. Before initiating the study, all participating sites received institutional review board approval. Written informed consent was obtained from all study participants before any study-related procedures were performed.

## Results

### Study Participants

Of the 203 randomized patients across both pivotal trials, 196 patients were included in the mITT population and used for this analysis (14, 15). Baseline characteristics of the mITT are summarized by treatment group in Table 1; treatment groups were well-balanced within and across studies (21). The mean age was 64.7 years (range, 34–82) and the mean time since PD diagnosis was 9.7 years (range, 1.0–26.8). On average, patients had been receiving levodopa therapy for a mean of 7.7 years (range, 1.1–20.3), and had been experiencing LID for a mean of 3.8 years (range, 0.1–14.0).

**Table 1:** Baseline demographic and PD characteristics, by treatment group (mITT population) (21).

**Variable**	**Gocovri**	**Placebo**
	**(*n* = 100)**	**(*n* = 96)**
Age (years), mean ± SD	64.2 ± 9.5	65.3 ± 8.8
Sex, *n* (%) male	54 (54.0)	55 (57.3)
Race, *n* (%)		
White	96 (96.0)	89 (92.7)
Other	4 (4.0)	7 (7.3)
Time since PD diagnosis (years), mean ± SD	9.8 ± 4.7	9.7 ± 4.1
Duration of levodopa treatment (years), mean ± SD	7.8 ± 3.9	7.6 ± 4.1
Duration of LID (years), mean ± SD	4.0 ± 3.1	3.6 ± 2.5
Levodopa dose (mg), mean ± SD	819.1 ± 487.2	743.3 ± 493.5
Concomitant PD medications, *n* (%)		
Dopamine agonist	50 (50.0)	56 (58.3)
MAOB-inhibitor	43 (43.0)	43 (44.8)
COMT inhibitor^a^	37 (37.0)	37 (38.5)
Anticholinergic	2 (2.0)	5 (5.2)

a *Includes patients taking Stalevo (levodopa, carbidopa and entacapone combination)*.

### Diary Data Analyses

At baseline, patients (*n* = 196) experienced a mean ± SD of 2.8 ± 2.1 h/day of OFF time, 3.7 ± 2.7 h/day of ON time *without* dyskinesia, and 9.5 ± 3.2 h/day ON time with dyskinesia. ON time with dyskinesia comprised 4.9 ± 2.6 h/day of ON time with troublesome dyskinesia and 4.6 ± 2.7 h/day of ON time with non-troublesome dyskinesia.

**Figure 1:**
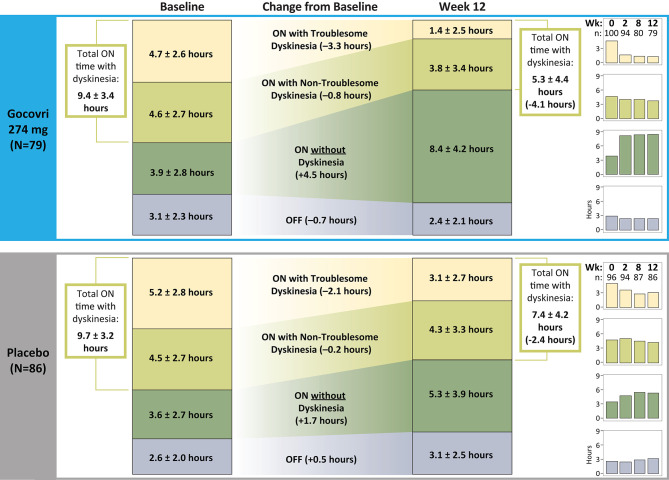
Change from Baseline to Week 12 in time spent in the different motor diary states (numbers are absolute mean ± SD).

As shown in Figure 1, patients randomized to receive Gocovri showed an increase in ON time *without* dyskinesia, from a mean ± SD of 3.9 ± 2.8 h/day at baseline to 8.4 ± 4.2 h/day at Week 12 (*n* = 79 with data at Week 12). Key drivers for increased ON time *without* dyskinesia were the reduction in ON time with troublesome dyskinesia together with the reduction in OFF time. Patients randomized to the placebo group also showed an increase in the time spent ON *without* dyskinesia, but this effect was primarily driven by a decrease in ON time with troublesome dyskinesia, whereas OFF time increased (from 2.6 ± 2.0 h/day at baseline to 3.1 ± 2.5 h/day at Week 12).

Consistent with the above results, time spent ON with dyskinesia (troublesome + non-troublesome) decreased in the Gocovri group from 9.4 ± 3.4 h at baseline to 5.3 ± 4.4 h at Week 12, whereas in the placebo group, total time with dyskinesia decreased from 9.7 ± 3.2 h at baseline to 7.4 ± 4.2 h at Week 12. The proportion of ON time with dyskinesia that was rated as troublesome (vs. non-troublesome) also showed a greater reduction for Gocovri, from 50% of total dyskinesia time at baseline to 26% at Week 12, compared with 54% at baseline and 42% at Week 12 for placebo.

Treatment effects were statistically significant for Gocovri vs. placebo starting at Week 2 for ON time with dyskinesia, ON time *without* dyskinesia, and ON time with troublesome dyskinesia, and statistically significant starting at Week 8 for OFF time (Table 2).

**Table 2:** MMRM Analysis of PD Diary Outcomes: LS Mean and Placebo-Adjusted Change from Baseline by Study Visit.

**Diary state (hours per day);**	**Placebo^a^**	**Gocovri^b^**	**Treatment difference**	***P*-value**
**LS mean ± SE change from baseline**				
ON *without* (*any)* dyskinesia^c^				
Week 2	1.2 ± 0.4	4.3 ± 0.4	3.2 ± 0.5	< 0.0001
Week 8	1.8 ± 0.4	4.5 ± 0.4	2.7 ± 0.6	< 0.0001
Week 12	1.7 ± 0.4	4.6 ± 0.4	2.9 ± 0.6	< 0.0001
ON with dyskinesia				
Week 2	−1.2 ± 0.4	−3.7 ± 0.4	−2.6 ± 0.5	< 0.0001
Week 8	−2.1 ± 0.4	−4.0 ± 0.4	−1.9 ± 0.6	0.001
Week 12	−2.2 ± 0.4	−4.1 ± 0.4	−1.9 ± 0.6	0.002
ON with non-troublesome dyskinesia				
Week 2	0.4 ± 0.3	−0.5 ± 0.3	−0.9 ± 0.4	0.02
Week 8	0.1 ± 0.3	−0.5 ± 0.3	−0.6 ± 0.5	0.24
Week 12	−0.3 ± 0.3	−0.8 ± 0.3	−0.6 ± 0.4	0.2
ON with troublesome dyskinesia				
Week 2	−1.4 ± 0.3	−3.3 ± 0.3	−1.8 ± 0.4	< 0.0001
Week 8	−2.1 ± 0.3	−3.6 ± 0.3	−1.5 ± 0.4	0.0001
Week 12	−1.9 ± 0.3	−3.3 ± 0.3	−1.5 ± 0.4	0.0003
OFF				
Week 2	−0.2 ± 0.2	−0.5 ± 0.2	−0.3 ± 0.2	0.17
Week 8	0.2 ± 0.2	−0.5 ± 0.2	−0.7 ± 0.3	0.01
Week 12	0.4 ± 0.2	−0.6 ± 0.2	−1.0 ± 0.3	0.0006

a *Placebo sample size: Week 2, n = 94; Week 8, n = 87; and Week 12, n = 86*.

b *Gocovri sample size: Week 2, n = 94; Week 8, n = 80; and Week 12 n = 79*.

c *ON without (any) dyskinesia is defined as ON time with neither troublesome nor non-troublesome dyskinesia*.

On MMRM analysis at Week 12, patients in the Gocovri group showed a LS mean ± SE increase over placebo (treatment difference) of 2.9 ± 0.6 h in ON time *without* dyskinesia (Cohen D effect size 0.79). Patients treated with Gocovri showed a LS mean ± SE reduction over placebo of −1.9 ± 0.6 h in ON time with dyskinesia (Cohen D effect size 0.49), including a −1.5 ± 0.4 h placebo-adjusted reduction in ON time with troublesome dyskinesia and a reduction in ON time with non-troublesome dyskinesia of −0.6 ± 0.4 h. As previously reported, Gocovri treatment also significantly reduced OFF time by an adjusted mean of −1.0 ± 0.3 h vs. placebo. Taking the LS mean treatment differences over the mean baseline score for all participants, these changes represented an ~79% placebo-adjusted increase in ON time *without* dyskinesia (including a 29% increase in ON time without troublesome dyskinesia), and corresponding 30 and 36% reductions in ON time with troublesome dyskinesia and OFF time, respectively.

### Thresholds of Response

**Figure 2:**
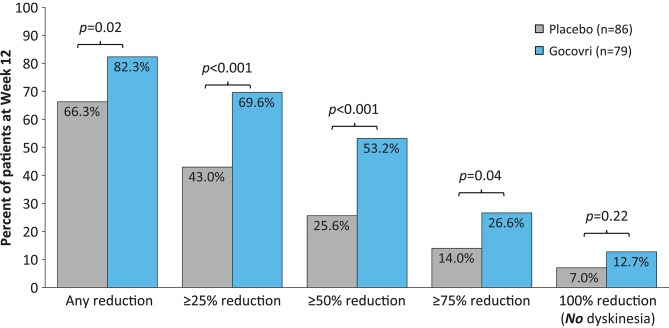
Distribution of patients achieving ≥25%, ≥50%, ≥75%, and 100% reductions in ON time with dyskinesia (both troublesome and nontroublesome). Two-sided *p*-values were obtained *post-hoc* using the Farrington-Manning (FM) score test for H0: Delta = 0, where Delta is the treatment difference in responder rates.

**Figure 3:**
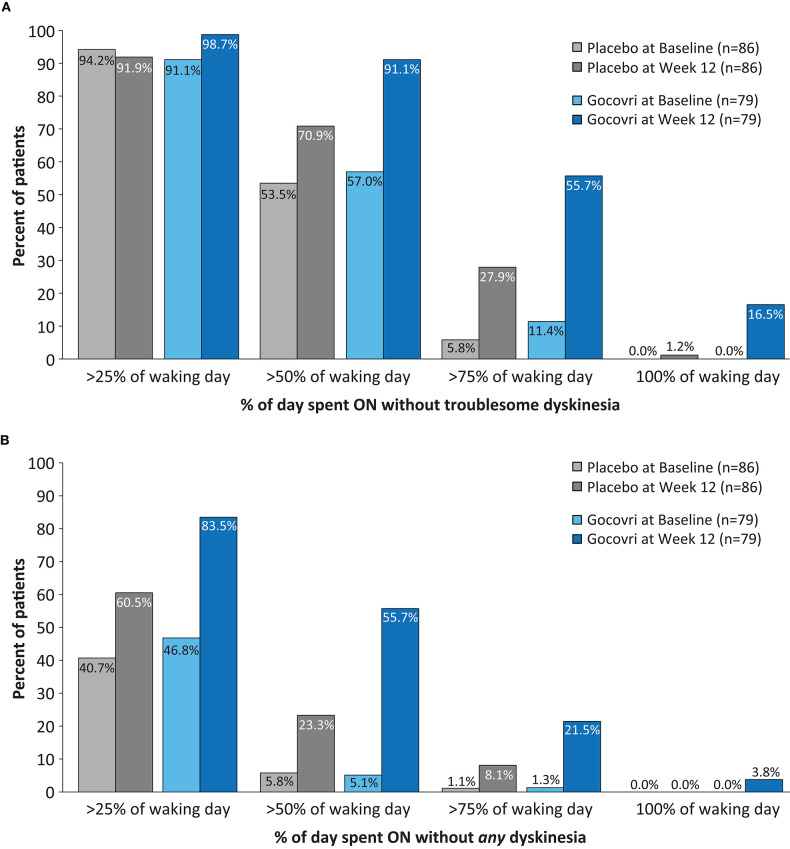
Proportions of subjects at Baseline and at Week 12 achieving >25, >50, >75, and >100% of time during the Waking Day **(A)** ON without troublesome dyskinesia (*p* < 0.001 favoring Gocovri vs. placebo) **(B)** ON *without* (troublesome or non-troublesome) dyskinesia. (*p* = 0.003 favoring Gocovri vs. placebo). Two-sided *p*-values comparing Gocovri vs. placebo in terms of the frequency distribution of shifts from baseline to week 12 (where each shift was categorized in one of nine ordered classes representing levels of improvement, worsening, or no change, were obtained using the Cochran-Mantel-Haenszel chi-squared test. The frequency distribution for the shifts was determined by using the cross-classification of subjects at baseline and at week 12 with % of time of 25, 50, 75 and 100%. The baselines for all subjects [N = 96 placebo and N = 100 Gocovri] were: A) >25%: 94.8% and 93.0%; >50%: 55.2% and 57%; >75%: 6.3% and 12.0%; 100% none B) >25%: 40.6% and 46.0%; >50%: 6.3% and 5.0%; >75%: 1% and 1%; 100% none.

The distribution of patient response by treatment group is shown in Figures 2, 3. Just over half (53%) of patients treated with Gocovri had ≥50% reduction in time spent ON with dyskinesia (troublesome + non-troublesome) and just over quarter (27%) of patients had ≥75% reduction compared with 26 and 14% of patients who received placebo, respectively. These differences were statistically significant based on *post-hoc* analysis. Of note, 13% of patients treated with Gocovri had no recorded ON time with dyskinesia at week 12, vs. 7% in the placebo group.

Significantly more patients treated with Gocovri experienced positive shifts in the percentage of the waking day spent ON without troublesome dyskinesia (*p* < 0.001) and (ii) in waking day ON *without* dyskinesia (*p* = 0.003) vs. placebo. At Week 12, over half (56%) of Gocovri-treated patients spent >75% of the waking day ON without troublesome dyskinesia compared with 28% of patients in the placebo group (Figure 3A). The percentage with *no* reported ON time with troublesome dyskinesia at endpoint was 16% for Gocovri compared with 1% for placebo. Likewise, the proportion of Gocovri-treated patients spending ≥50% of their waking day ON *without* dyskinesia rose from 5 to 56% at Week 12—compared with a corresponding increase from 6% to 23% in the placebo group (Figure 3B).

## Discussion

To our knowledge, we present the first analysis of contemporary clinical trial diary data focusing on time spent ON *without* dyskinesia (either troublesome or non-troublesome) as an outcome. At Week 12, participants taking Gocovri showed a significant LS mean increase of 4.6 h in the daily duration of total ON time *without* any dyskinesia, a treatment difference of 2.9 h over placebo (95% CI: 1.83, 4.07; *P* < 0.0001). Improvement with Gocovri was mainly due to a robust decrease in time spent ON with troublesome dyskinesia (reduction of 1.5 h/day over placebo) combined with a similarly robust decrease in OFF time (reduction of 1.0 h/day over placebo). Antidyskinetic effects were seen as early as the first on-treatment follow-up visit (2 weeks) and were sustained at subsequent visits. Responder analyses highlighted that just over half of patients had at least a 50% reduction in time spent on with dyskinesia, and for over one in ten patients, dyskinesia was completely resolved.

Patients and caregivers often dislike the observable hyperkinetic movements of dyskinesia and the stigma that may accompany it (2, 3). Since most adjunctive treatments for OFF tend to increase dyskinesia, clinical trials typically set a goal of attempting to decrease OFF time without increasing troublesome dyskinesia (i.e., dyskinesia that interferes with function or causes meaningful discomfort) and may accept some increase in non-troublesome dyskinesia (22, 23). Similarly, in clinical trials of anti-dyskinetic medications, investigators generally aim to decrease troublesome dyskinesia without worsening OFF time. In both of these cases, we are attempting to increase ON time without troublesome dyskinesia. As highlighted by the present analyses, treatment with Gocovri was able to decrease both ON time with dyskinesia (troublesome + non-troublesome) and OFF time, including a meaningful increase in the percentage of patients *without* dyskinesia. In this case, while the primary emphasis may still be on the traditional goals of decreasing OFF time and ON time with troublesome dyskinesia, additional benefit may potentially be derived by also decreasing ON time with non-troublesome dyskinesia—so long as it can be achieved without increasing OFF time. Diaries may not completely reflect all potential dysfunction during patient-defined non-troublesome dyskinesia as the dysfunction (troublesomeness) may only be present for a minority of the time period. Further, patients may not fully recognize their LID, including its functional impact (24–26). The reduction in ON time with non-troublesome dyskinesia may also be important to provide a buffer that allows clinicians to increase levodopa to further decrease OFF time and improve parkinsonian symptoms without increasing ON time with troublesome dyskinesia.

Strengths of the present analyses lie in the similar design and conduct of the two pivotal studies which allowed prespecified pooling of the data to provide more precise estimates of the treatment difference (Gocovri minus placebo) than could be reported in the separate studies. It is also relevant that both studies assessed the impact of Gocovri treatment in the “target population”, namely those PD patients with clinically relevant levels of LID at baseline. On the other hand, the LID-based inclusion criteria may limit generalizability for patients with only non-troublesome LID. Other important limitations should also be acknowledged. While each of the different motor states were prospectively collected in each of the studies, the responder analyses were performed *post-hoc* (although appropriate statistical measures were utilized to address this). The patient diaries are designed to record the predominant state during each 30-min diary interval. Therefore, we are not able to state with certainty that 30-min blocks reported as ON *without* dyskinesia translate to a complete absence of dyskinesia vs. predominance of ON *without* dyskinesia. Likewise, patients only captured 2 days of diary data prior to each clinic visit, which may not be entirely reflective of typical daily or weekly experiences. Continuous monitoring with wearable devices may provide a better measurement of dyskinesia presence once technology advances are able to reliably accommodate this and they are appropriately validated (27).

Although effects were clearly sustained from Week 2 through to Week 12, longer study durations are required to confirm maintenance of effect. In the longer EASE LID study, treatment benefits in increasing ON time without troublesome dyskinesia, and reducing OFF time, time spent ON with troublesome dyskinesia and with dyskinesia (troublesome + non-troublesome) were maintained and were all significantly different than placebo (all *p* < 0.05) among those patients who completed the entire 24 weeks of treatment (before the study was prematurely stopped to allow regulatory filing) (14). Recent analyses of the open-label EASE-LID 2 study further showed maintenance of benefits in terms of reducing ON time with dyskinesia and OFF time (as assessed by the MDS-UPDRS Part IV) over 2 years (28). Finally, while patients in the trial had to have peak-dose dyskinesia, other forms of dyskinesia were not excluded, and combinations of choreic and dystonic movements, peak-dose and end-of-dose dyskinesias often occur in the same patient. However, the various types of dyskinesia experienced are not able to be captured in current outcome measures and it would be of obvious interest to understand if treatment with Gocovri were helpful for these less common forms of dyskinesia.

In summary, results of these pooled analyses show a robust effect of Gocovri in increasing time patients spend ON *without* dyskinesia by reducing overall motor complications including time spent ON with both troublesome and non-troublesome dyskinesia as well as time spent OFF.

## Data Availability Statement

Where patient data can be anonymized, Adamas Pharmaceuticals Inc will share all individual participant data that underlie the results reported in this article with qualified researchers who provide valid research questions. Study documents, such as the study protocol and clinical study report, are not always available. Requests to access the datasets should be directed to info@adamaspharma.com.

## Ethics Statement

The studies involving human participants were reviewed and approved by an ethics committee. Copernicus was the IRB for each study. Additionally, each investigator was required to obtain institutional review board (IRB)/research ethics board (REB)/independent ethics committee (IEC) approval at their investigative site. Written approval of these documents was obtained from the IRB/REB/IEC before any subject was enrolled in the study at the investigational site. The patients/participants provided their written informed consent to participate in this study.

## Author Contributions

The original idea for this manuscript was conceived by RAH. RAH, DC, and AEF wrote the first draft of the manuscript. RAH, RW, and RP were investigators in the Gocovri pivotal trials. AEF led the analysis and manuscript oversight and provided critical review of the draft. All authors contributed to the analysis plan, interpretation of results, and approved the final version of the article.

## Conflict of Interest

RAH was supported in part by a Center of Excellence grant from the National Parkinson Foundation and is employed by the University of South Florida (Florida). He received payment from Adamas Pharmaceuticals, Inc. for participating as a Steering Committee member and reports receiving personal fees from Acadia Pharmaceuticals, Acorda therapeutics, Adamas Pharmaceuticals, Affiris, AlphaSights, Amneal Pharmaceuticals, ApoPharma, Aptinyx, Aranca, Axovant, Britannia, Cadent, CAVR, Cerevel Therapeutics, ClearView Healthcare Partners, Clinical Score LLC, CNS Ratings LLC, Compass Group, Decision Resource Group (DRG), Dedham Group, Defined Health, Denali, Enterin, Extera Partners, F. Hoffmann-La Roche Ltd., First Word, Gerson Lehman Group (GLG), Global Kinetics Consulting (GKC), Global Life Sciences, Guidepoint Global, Huron, Impax Laboratories, Impel Neuropharma, Inhibikase, InSearch Consulting, Insignia Strategies, In-Trace Medical Systems, ISCO, IQVIA, Jazz Pharmaceuticals, Kaiser Permanente, Kashiv Pharma, KeiferRX LLC, KeyQuest, KX Advisors, Kyowa Kirin Pharmaceuticals, L.E.K Consulting, LifeSciences Consultants, Lundbeck A/S, Medscape, MJFF, MPTA, Neuro Challenge Foundation for PD, Neurocrine Biosciences, NeuroDerm, NOVUS, Orion, Parkinson Study Group, Pennside Partners, Perception OpCo, Pharmather, PSL Group, Regenera Pharma, Revance Therapeutics, Schlesinger Associates, Scion NeuroStim LLC, Seelos Therapeutics, Slingshot Insights, Sunovion Pharmaceuticals, Supernus Pharma, Teva Pharmaceuticals, Tolmar, Inc., US World Meds. RAH reports research support from AbbVie, Axovant Sciences Ltd, Biogen Inc, Biotie Therapies Inc, Cavion, Inc, Centogene, Cerevance, Cerevel Therapeutics Inc, Cynapsus Therapeutics, Enterin Inc, F. Hoffman-La Roche Ltd, Global Kinetics Corporation (GKC), Impax Laboratories, Intec Pharma, Jazz Pharmaceuticals, Michael J Fox Foundation, Neuraly, NeuroDerm Ltd, Northwestern University, Pfizer, Pharma Two B, Revance Therapeutics, Sanofi US Services Inc, Sun Pharma Advanced Research Company, Sunovion Pharmaceuticals Inc and hold stock in Inhibkase and Axial Therapeutics. RW reports consulting compensation from Adamas, Acadia, Alexion, AbbVie, Bukwang, Clarion, Lundbeck, Prime, Sarepta, Syneos, Techspert, Teva, Wilson Therapeutics and Mosaic Research Management, and research funding support from Parkinson's Disease Foundation Postdoctoral Fellowship in Clinical Research, Civitan International Research Center Emerging Scholars Award, NIH-NIGMS Center of Biomedical Research Excellence P20GM109025, Elaine P. Wynn & Family Foundation Lee Pascal Parkinson's Disease Scholar, Sam and Peggy Grossman Foundation, and Samuel P. Mandell Foundation. RP reports consultancy for Acadia, Adamas, Impax, St Jude Medical, Teva Neuroscience, Medtronic, and US WorldMeds. He has received honoraria from Medtronic, Teva Neuroscience, UCB, and US WorldMeds. He has received research grants from Acadia, Adamas, Avid, NIH/NINDS, NPF, and PSG/University of Rochester. He has also served on the data monitoring committee for Ceregene. DC was employed by Adamas Pharmaceuticals, Inc. at the time of the analysis and manuscript preparation. AEF is employed by Adamas Pharmaceuticals, Inc.
